# How to set up a low cost tele-ultrasound capable videoconferencing system with wide applicability

**DOI:** 10.1186/2036-7902-4-13

**Published:** 2012-05-29

**Authors:** Innes Crawford, Paul B McBeth, Mark Mitchelson, James Ferguson, Corina Tiruta, Andrew W Kirkpatrick

**Affiliations:** 1 University of Aberdeen, Aberdeen, Scotland, AB24-3, U, United Kingdom; 2 Department of Surgery, Foothills Medical Centre, Calgary, Alberta, AB-T2N-2T9, Canada; 3 Department of Emergency Medicine, Aberdeen Royal Infirmary, Urquhart Road City Centre, Aberdeen, AB24 5AU, Scotland, UK; 4 Scottish Centre for Telehealth, Riverside Drive, Aberdeen, Scotland, AB11 7LH, UK; 5 Critical Care Medicine, Foothills Medical Centre, Calgary, Alberta, AB-T2N-2T9, Canada; 6 Regional Trauma Services, Foothills Medical Centre, Calgary, Alberta, AB-T2N-2T9, Canada

**Keywords:** Tele-ultrasound, Injury, Pre-hospital, Trauma systems

## Abstract

**Background:**

Worldwide ultrasound equipment accessibility is at an all-time high, as technology improves and costs decrease. Ensuring that patients benefit from more accurate resuscitation and diagnoses from a user-dependent technology, such as ultrasound, requires accurate examination, typically entailing significant training. Remote tele-mentored ultrasound (RTUS) examination is, however, a technique pioneered in space medicine that has increased applicability on earth. We, thus, sought to create and demonstrate a cost-minimal approach and system with potentially global applicability.

**Methods:**

The cost-minimal RTUS system was constructed by utilizing a standard off-the-shelf laptop computer that connected to the internet through an internal wireless receiver and/or was tethered through a smartphone. A number of portable hand-held ultrasound devices were digitally streamed into the laptop utilizing a video converter. Both the ultrasound video and the output of a head-mounted video camera were transmitted over freely available Voice Over Internet Protocol (VOIP) software to remote experts who could receive and communicate using any mobile device (computer, tablet, or smartphone) that could access secure VOIP transmissions from the internet.

**Results:**

The RTUS system allowed real-time mentored tele-ultrasound to be conducted from a variety of settings that were inside buildings, outside on mountainsides, and even within aircraft in flight all unified by the simple capability of receiving and transmitting VOIP transmissions. . Numerous types of ultrasound examinations were conducted such as abdominal and thoracic examinations with a variety of users mentored who had previous skills ranging from none to expert. Internet connectivity was rarely a limiting factor, with competing logistical and scheduling demands of the participants predominating.

**Conclusions:**

RTUS examinations can educate and guide point of care clinical providers to enhance their use of ultrasound. The scope of the examinations conducted is limited only by the time available and the criticality of the subject being examined. As internet connectivity will only improve worldwide, future developments need to focus on the human factors to optimize tele-sonographic interactions.

## Background

Ultrasound is a portable (often hand or pocket-carried) noninvasive, cost-effective, multidisciplinary/multisystem diagnostic and resuscitative tool that has potential applicability in nearly every aspect of patient care [1-3]. Tele-ultrasound is a specific form of telemedicine that uses informatics advances to separate the patient from the expert interpreting the ultrasound findings, using techniques that are supported through teleconferencing technology [4]. The teleconferencing equipment to facilitate this is currently ‘big business’ as many health services in developed countries invest in it for the future. Videoconferencing (VC) equipment, however, can be notoriously expensive and can involve large start-up costs that discourage many health care systems as does the personal time commitment needed to respond to a call considering individual healthcare providers. Costs have been identified as one of the main barriers to the implementation of telemedicine, particularly for developing countries who would likely benefit the most from this technology [5].

Other informatics advances, however, are furthering the revolution in connectivity and drastically reducing costs and logistic requirements. Voice over internet protocol (VOIP) allows voice and other multimedia (e.g., video) to be transmitted *via* the internet instead of over other means like the public-switched telephone network (PSTN, normal telephone) or over Integrated Service and Digital Network (ISDN) lines that are traditionally used for videoconferencing. VOIP is bandwidth efficient and has low running costs prompting many businesses including hospitals to migrate from using the traditional telephone to VOIP [6]. VOIP can also utilize cellular networks as well as wireless or Ethernet links, allowing access wherever there is coverage. VOIP is also capable of linking into PSTN and mobile network providers allowing calls to these services [7]. Examples of VOIP providers include Apple's Facetime (Apple Inc., USA), Google's Chat (Google Inc., CA, USA), Oovoo of New York (Oovoo LLC, GA, USA), and one of the most widely used, Skype (Skype Technologies S.A., Luxembourg, Luxembourg). Skype is a free VOIP service that currently provides low cost VC services to millions of people and businesses worldwide. With the continuing development of VOIP services, providers like Skype now offer many new services including group video calling, high-definition (HD) videoconferencing and 3 G video calling [8]. VOIP, in particular Skype, has been investigated by several authors for use in healthcare mainly as a way of replacing older PSTN systems in hospitals to reduce costs [6]. It has also been investigated as a way of providing teaching in geographically difficult locations [9] and for providing support in remote locations such as an Amazon swim expedition [10] and for assessing burn victims in Sao Tome [11]. While innovative, these prior applications only evaluated VOIP capabilities to provide basic video calling and not for the transmission of other more robust forms of medical informatics. Recognizing a clinical need in both our own referral area, as well as globally, we thus demonstrated the ability to construct a low cost tele-ultrasound capable VC setup solution for the transmission of medical informatics over Skype [12,13].

## Methods

### System setup

**Figure 1:**
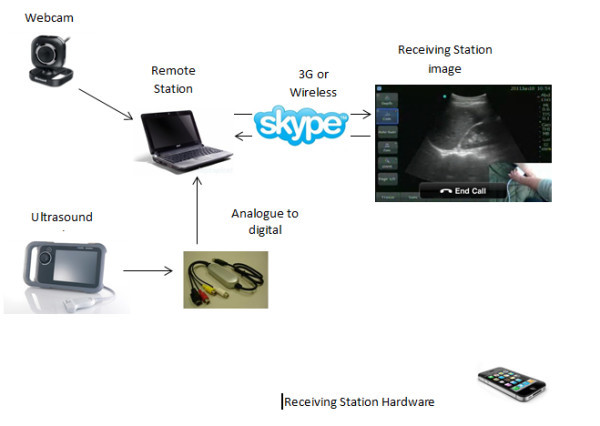
Schematic overview of the system design paradigm.

The freely available VOIP software Skype (5.1) was installed onto modestly priced commercially available laptop computers (Acer Travelmate 5520 and Aspire 5741, Acer Inc., Kuala Lumpur). A second, freely available software called XSplit Broadcaster (SplitMediaLabs Ltd., Hong Kong) was concurrently installed that allowed the display of several video inputs either simultaneously or independently by creating a virtual webcam that could thereafter be selected as a video output on Skype. An inexpensive analogue to digital converter (VC-211 V, ActionStar LinXcel, Taiwan) connected a modestly priced hand-carried ultrasound machine (NanoMaxx, Sonosite Inc., Bothell, WA) to the laptop. A head-mounted webcam (LifeCam VX-2000, Microsoft Corporation, Washington) was also connected to the base-station laptop to provide real-time images of the patient and position of the ultrasound probe. Detailed ‘step-by-step’ instructions on how to construct such a network can be seen in Additional file 1. A schematic overview of the system design paradigm is presented in Figure 1.

## Results

### System evaluation

A number of demonstrations verified the ability of this approach to facilitate remote mentored telesonography between geographically separate caregivers located on different continents, such as across the Atlantic between Calgary and alternatively Aberdeen, Scotland, and Rome and Pisa, Italy and in different remote environments, including from within small airplanes and from mountain-side locations [12,14]. These are summarized in Table 1. Detailed instructions on how to initiate such tele-ultrasound sessions over Skype as an example of a VOIP can be seen in Additional file 2. Although many remote mentored sessions using this particular system have been undocumented, formally documented sessions to the time of writing are summarized in Table 1 and briefly elaborated on below.

**Table 1:** Formally documented low-cost tele-ultrasound remote mentoring reports

**Author**	**Number of examinations**	**Site of examination**	**Experience of on-site user**	**Site of interpretation**	**Focus of examination**
McBeth [12]	1	Mountain, Canada	Basic	House, Canada	EFAST exam [15]
Crawford [16]	1	Hospital, Canada	Novice	Hospital, Scotland	Lung ultrasound
Crawford [13]	1	Hospital, Canada	Novice	Hospital Scotland	FAST exam
McBeth [14]	10	Varied settings in Canada	Novice	Varied including international Trans-Atlantic	Lung ultrasound
Biegler [17]	1	Hospital, Canada	Novice	Same hospital, Canada	Lung ultrasound post-chest tube
Biegler (unpublished)	13	Hospital	Basic to novice	Same hospital, Canada	Lung ultrasound post-chest tube

EFAST, extended focused assessment with sonography for trauma; FAST, focused assessment with sonography for trauma.

Once such a system has been constructed to transmit from the site of the patient as a ‘package’ delivered over VOIP, the receiving station may be as varied as there are computer or smartphone devices that will support a particular VOIP software. This freedom allows the remote mentor to be able to use any of their desktop computer, laptops, or smart devices such as smartphones or tablet-type devices. For example, a video call was initiated by the user in Calgary, Canada to a panel of remote experts in Aberdeen, Scotland. The senders were connected to a 3 G network, facilitated by the use of a ‘dongle/rocket stick’ (Rocket Mobile Internet Stick, Nokia Corp., Keilaniemi, Finland) to facilitate the connections between the laptops. A rocket stick is essentially a device that allows the laptop to connect to the internet through the same way your smartphone does when not connected to Wifi. Your smartphone and tablet computers normally have this built in, and you can in fact use some mobiles themselves as a ‘rocket stick’. The remote experts guide a user through both a basic Focused Assessment with Sonography for Trauma (FAST [18]) performed on an ultrasound phantom and a volunteer, interpreting the images as they were captured [13]. The extended FAST exam [18] examining the pleural spaces was also performed and demonstrated remotely [16] [see Additional file 3.

## Discussion

The current generation of the cost-minimal RTUS system reflects a rapid but continuous evolution of technology designed to increase the accessibility and decrease the infrastructure required, ultimately increasing affordability. While there were notable prior efforts to develop emergency remote tele-ultrasound capabilities [19,20], the development of remote *mentored* tele-ultrasound was led by investigators from or supported by the National Aeronautics and Space Agency (NASA), charged to develop medical support capabilities for the crew members of the International Space Station [21,22]. The first real-time terrestrial trials of this approach in actual injured patients were subsequently performed between Banff and Calgary in Alberta using dedicated internet lines [23,24]. This work demonstrated the practicality of real-time acute trauma RTUS, but noted logistical challenges in sustainability. Various efforts to increase the portability of RTUS have involved simplifying the equipment required and portability. Dulchavsky and colleagues have, thus, championed the use of a stand-alone video compression device to stream through a secure satellite modem, thus allowing uni-directional ultrasound and video transmissions with bidirectional audio from locations such as Mount Everest and the Canadian Arctic [4,25,26]. In a similar manner, remote musculoskeletal and thoracic examinations for high altitude pulmonary edema and joint examinations by novices were guided from Henry Ford Medical Centre [25,26].

Our own efforts in Calgary, Alberta have attempted to simplify RTUS even further, using freely available VOIP software to transmit and provide remote mentors with ultrasound images produced by the novice in conjunction with simultaneous real-time views of the novices handling of the ultrasound probe. Once the macro scene and ultrasound images have been assembled and transmitted using VOIP, the remote mentor can be view the examination upon any electronic device that receives a password-protected secure internet signal such as desk or laptop computer, a tablet device, or a smartphone.

The system setup as described is both easy to implement and low cost, allowing the remote user to view both the ultrasound image being captured and the base user. Such a system allows any remote content expert to guide a novice through a basic ultrasound scan and to ultimately augment clinical diagnoses with this information. In order for administrators, researchers, and clinicians to fully appreciate the potential of such an approach, there are several areas that warrant further discussion regarding the use of VOIP, specifically the use of Skype in this manner namely: security and privacy, image, audio quality, connectivity, and the scope of potential applications.

### Security and privacy issues

Concerns are intuitively generated regarding the transmission of patient data over the internet using a third party, especially as Skype which is a proprietary ‘closed-source’ software, making it difficult to objectively assess its security [27]. Health-related data transmission in Canada must meet the Personal Information Protection and Electronic Documents Act and the Health Information Privacy Code standards [28]. Skype transmissions are protected by multiple systems to guarantee security and privacy. All information is sent over secure socket layer that uses 256-bit Advanced Encryption Standard (AES) for all the information, leaving a transmitting computer that can only be decrypted by the Skype server. This same technology protects online bank users when transactions are made over the internet. User information is further encrypted through the required password-protected sign-in that further protects against malicious third parties. Skype also uses digital certificates that are issued to everyone using Skype, providing assurance that a particular Skype account can only be used by the password holder and stops third parties impersonating them. Finally, Skype is also compatible with and can work through firewalls, allowing further protection from potentially malicious third parties [29]. Despite these multiple safeguards, there are legitimate concerns about data security on Skype [27].

Practically, it has been argued that Skype is invariably more secure than traditional phone networks that are already used to convey information to patients and other health professionals, yet they are traditionally very easy for third parties to tap into. The potential difference is that access with a software such as Skype involves a potential worldwide network, and with unauthorized access, it is also not just the transmission that is vulnerable but the computers themselves. We believe the ultimate solution to this potential risk is to continue to develop secure VOIP networks that many large health networks including both the Alberta Health Services and the National Health Services are currently designing and implementing [30]. These networks will likely require development and integration time to develop to the same level of sophistication and ease of use as current systems like Skype.

### Image and audio quality

There are very few studies that look specifically at ultrasound image quality transmission over 3 G networks or the internet with the few studies that are available being out of date with current transmission standards. However, several previous tele-ultrasound studies conducted using commercial VOIP systems with 3 G and low-bandwidth (256 kbit/s) internet transmission ultrasound images still concluded, however, that even with decreased image quality, the images were still of diagnosable quality [31,32].

There are several critical issues regarding video quality during video calling that ultimately determine the quality of the ultrasound and any accompanying video display. Skype automatically displays video as 320 × 240 pixels over the 3 G networks that is equivalent to the screen on a Nintendo DS (Nintendo, Redmond, Washington) or Sonosite 180 ultrasound (Sonosite Inc.). This is due to the increased bandwidth required for displaying images in 640 × 480 pixels and above (similar to an analogue computer screen). It is possible to display images at 640 × 480 if you are connected *via* WiFi at both ends, and Skype will now support high definition (1,280 × 720 pixels up to 30 fps) if transmitting over bandwidths of greater than 1 Mbps and an HD webcam is available [8]. These advances, thus, open up whole new areas of potential applications such as potentially allowing virtual teams or networks, with for instance, surgical and medical clinicians, radiologists, and pathologists, among many other experts simultaneously mentoring and guiding a remote inexperienced clinician through a developing critical case. While current videoconferencing equipment has this very capability, it can involve large start-up costs both for the equipment and setting up of ISDN lines, but if Skype or other VOIP systems could be used, drastically decreased costs would be involved to provide similar quality transmissions.

In addition to the added informatics requirements of two-way tele-communication, the present system also incorporated multiple video inputs into the sending video stream (ultrasound and video display, as well as an optional macro-scene video display). While it has been queried as to whether there will be degradation in image quality related to the transmission of two or more video feeds simultaneously, this criticism is not pertinent to the current system. This is related to the nature of the software, such as XSplit Broadcaster (Splitmedialabs, Ltd.), that actually combines two or more video inputs from the laptop computer to ultimately be selected as a single video output by Skype, potentially limiting this image degradation.

### Connectivity

A marked advantage of VOIP providers such as Skype is that they can be supported by either 3 G and wireless networks allowing maximum portability. Both approaches have been evaluated by this team and found to be practical. Nonetheless, this report noted that wireless afforded greater image and voice quality as well as being more reliable with less frequent dropped calls or compromised video quality. However, for sheer mobility and convenience of access, the 3 G signal performs well and was still able to provide images of diagnosable quality. Furthermore, with the development of 4 G handsets and the imminent launch of 4 G network services, quality and reliability will further improve with the image quality only continuing to improve with time and development.

### Applications

While this system and paradigm was tested in one of the most developed countries, one of the greatest areas of potential use for this system would be in the developing world. Telemedicine has been identified by the World Health Organization (WHO) as the potential to bring significant benefit to developing countries [5]. However, this great potential is tempered by concerns regarding perceived high costs, lack of technical support, and limited availability of equipment [5]. WHO reports also highlight, however, the potential of internet-based conferencing for addressing these issues, particularly mentioning a low-cost internet-based VC system used to provide maternal and newborn support in Mongolia including prenatal ultrasound diagnostics [5]. Other countries are also beginning to see the benefits of VOIP. In the US, several psychiatrists now perform regular consultations over Skype, and in Russia, Skype is used to transmit live operations and radiological images to allow remote senior clinical support [33].

There are almost unlimited areas of potential future application in both developing and developed countries. Medical education has already been investigated by Okrainec et al. [9] who demonstrated the advantages of a low-cost system using Skype to train surgeons in Botswana on basic laparoscopic techniques. These techniques could be extrapolated to provide medical education anywhere in the world, from anywhere in the world, the opportunity to provide top quality education to underserved countries. The very same tele-ultrasound system discussed currently very easily provides two-way teleconferencing even without using ultrasound, and we have used in our operating rooms to allow virtual surgical second opinions.

Prehospital care is fraught with immediate but critical decisions that are required of prehospital providers with variable skills and experience. Thus, another area of potential application would be within ambulances. Technically, this should be feasible as many ambulances in Canada already have portable laptops, 3 G access and video cameras installed. Many already transmit ECG images for remote support, and it could potentially be a simple matter of combining the already available equipment to allow real-time video support. We, thus, speculate that ultimately simplified tele-ultrasound systems could dramatically help on-call clinicians by replacing the traditional phone call with bidirectional audio and visual communication, allowing remote viewing of US images, patient information and even patients themselves, thus improving decision making and support, all from the accessibility of their personal laptop or smartphones.

## Conclusions

Skype can transmit video, audio and other data, providing remote access to wherever an internet signal of 3 G or better is available, and worldwide internet connectivity will only get better. We, thus, envision simplified methods of both improving the accuracy of clinical diagnosis and facilitating on-going medical education as being applicable worldwide wherever internet connectivity is available, but especially in settings where there are no other imaging resources.

## Competing interests

The authors declare that they have no competing interests.

## Authors' contributions

IC, PBM, MM, JF, and AWK conducted the literature review and conceived of the program of studies. IC, CT, PBM, and AWK wrote the grants and ethics applications to conduct the research. IC, PBM, MM, JF, CT, and AWK performed the research and analyzed and interpreted the findings. IC, PBM, CT, and AWK drafted the related manuscripts. IC, PBM, MM, JF, CT, and AWK critically reviewed all manuscripts. All authors read and approved the final manuscript.

## Supplementary Material

Additional file 1 **Title: How to set up Skype US system.** Description: Detailed ‘step-by-step’ instructions on how to construct a network (http://www.traumacanada.org/Default.aspx?pageId=829763) Click here for file

Additional file 2 **Title: Making a video call.** Description: Detailed instructions on how to initiate such tele-ultrasound sessions over a VOIP such as Skype (http://www.traumacanada.org/Default.aspx?pageId=829763). Click here for file

Additional file 3 **Title: Additional file 3.** Description: Tele-ultrasound recipe for the gourmets and aficionados (http://www.traumacanada.org). Click here for file
