# Supplementary material for: How to set up a low cost tele-ultrasound capable videoconferencing system with wide applicability
Source: Crit Ultrasound J. 2012 May 29;4(1):13. doi: 10.1186/2036-7902-4-13 (PMC3447660; doi:10.1186/2036-7902-4-13)
Supplement: Additional file 1 — Title: How to set up Skype US system. Description: Detailed ‘step-by-step’ instructions on how to construct a network (http://www.traumacanada.org/Default.aspx?pageId=829763) [file 2036-7902-4-13-S1.pdf]

How to setup Skype US system

1. **First Time Setup**
   a. Install latest version of Skype
      i. Download and install Skype, either the business or regular version, http://www.skype.com/intl/en-us/get-skype/
      ii. Open up and create an Account, then add contacts who you might phone
      iii. Do this by going to *‘Contacts’* and *‘Add a Contact’*, you will need their Skype name to be able to call them
   b. Install XSplitBroadcaster and Setup
      i. Create account at http://www.xsplit.com/ and download and install XSplit
      ii. Open up XSplit and go to *‘Tools’* and *‘Settings’*
      iii. Select the *‘General’* Tab
      iv. Under ‘Interaction’ ensure the *‘Enable virtual camera output’* and *‘Enable Skype interaction’* boxes are ticked, then click *‘Apply’* and *‘OK’*
   c. Select XSplit as Skype webcam
      i. Open up Skype again
      ii. Go to the *‘Tools’* tab and select *‘Options…’*
      iii. Under the *‘General’* tab select *‘Video Settings’*
      iv. Click on the *‘Select Webcam:’* drop box and select ‘XSplitBroadcaster’ then click *‘Save’*.
      v. If XSplitBroadcasterdoesn’t display as one of the options restart your computer and ensure XSplit is open before opening up Skype, it should display then

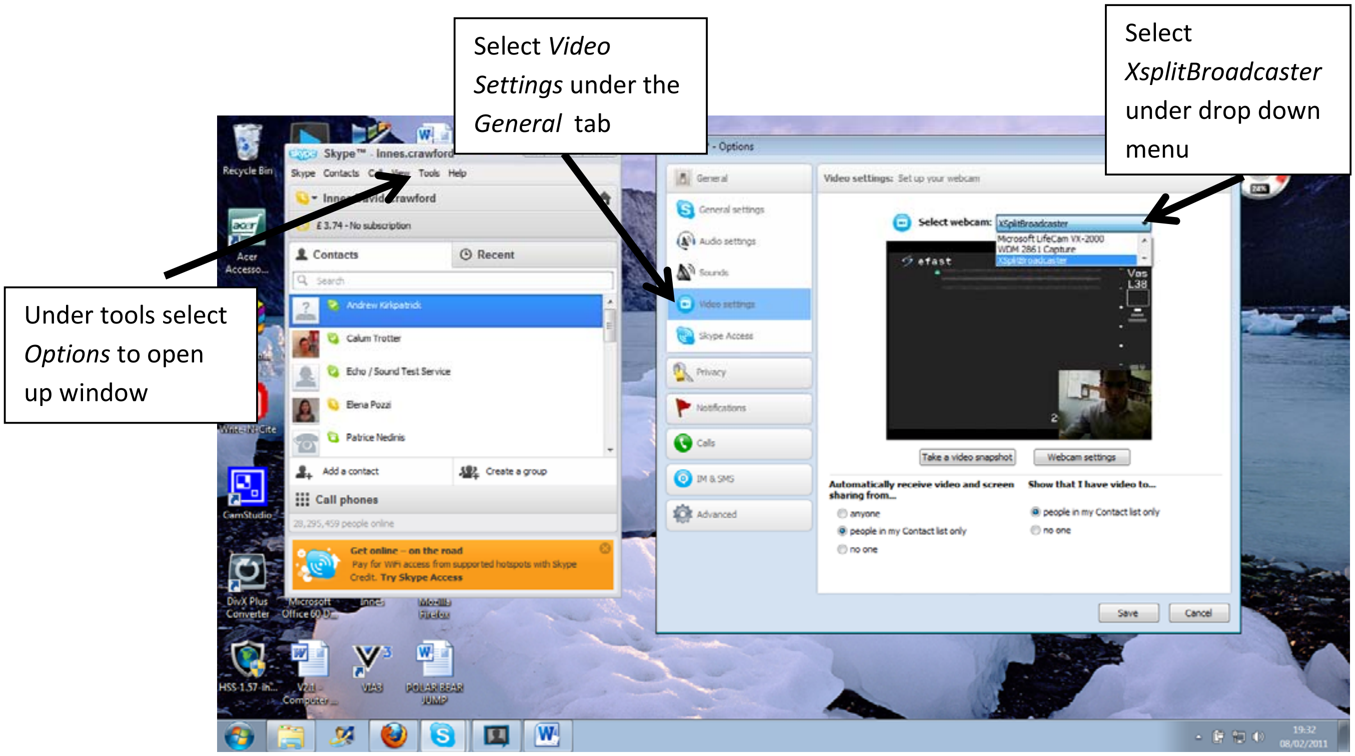

d. Setup of Equipment

  i. Plug Headcam into USB port on computer

    1. If first time, may need to install driver, laptop should do this automatically or guide you through it

  ii. Plug in VC-211V (videocapture device) into laptop and plug into Video output on ultrasound machine

e. Select Output on XSplit

  i. There are twelve scene boxes on the bottom right, select '*Scene 1*'

  ii. On the bottom right it says 'Add' click on that and click on *'Add Camera'* select '*WDM2861 Capture'*(or whatever your videocapture device is called)

  iii. It will then open up a screen displaying your US machine output, you can resize this to fit the whole screen

  iv. Click on *'Add'* and *'Add Camera'* again and select your webcam, this will again open up the output of your Webcam which you can then resize to the appropriate place, I normally make it smaller than the US image and display it on the bottom right as seen in Figure 1.

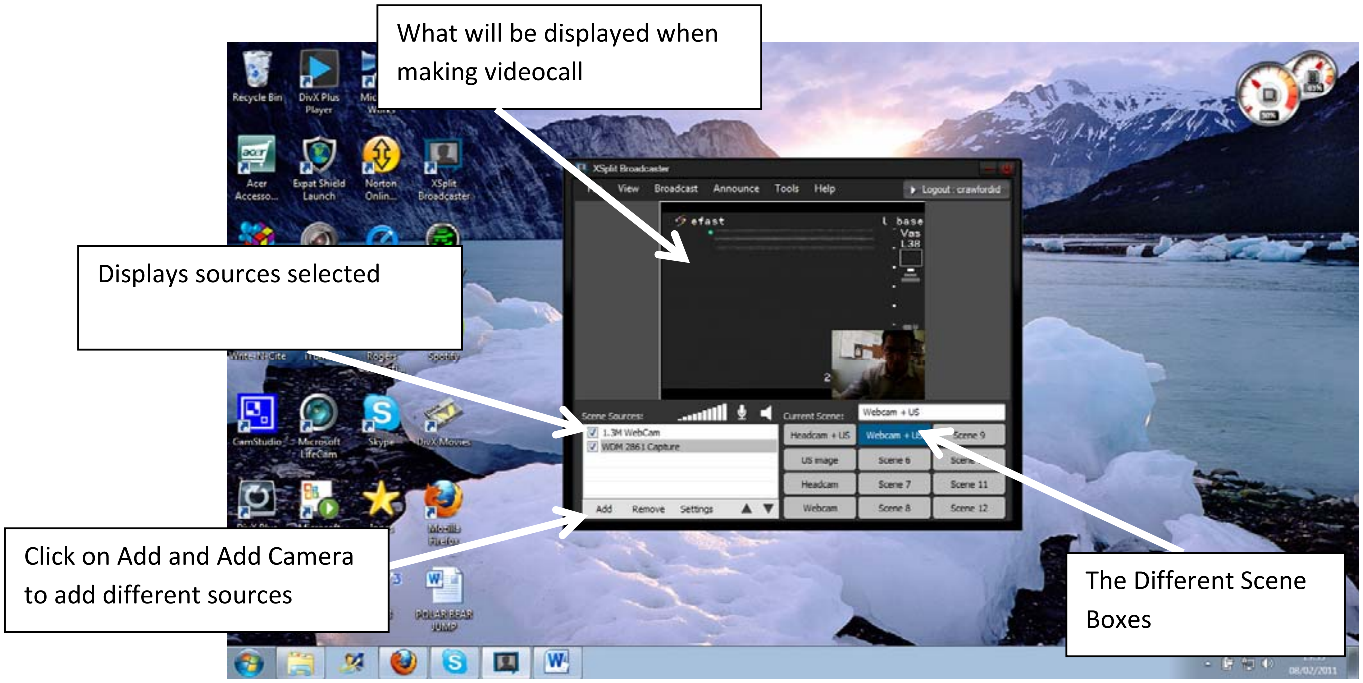

f. Extra Settings on XSplit,

   i. Different Scenes can display different outputs i.e. just enlarged webcam or just US image, just select the scene and add different cameras

   ii. You can change the name of the scenes i.e. Headcam + US

   iii. You can also change the hotkeys or use their existing ones to switch between Scenes (Outputs), I normally have F1 setup as US + Headcam and F2 as just US image and F3 as just the Webcam image

   iv. You can change various Output settings of XSplit i.e. Resolution and Frame rate, you may want to do this depending on if you are connecting via Wifi or 3G, I have it set to 320x240 and 30fps, you can change this under the *'view'* tab

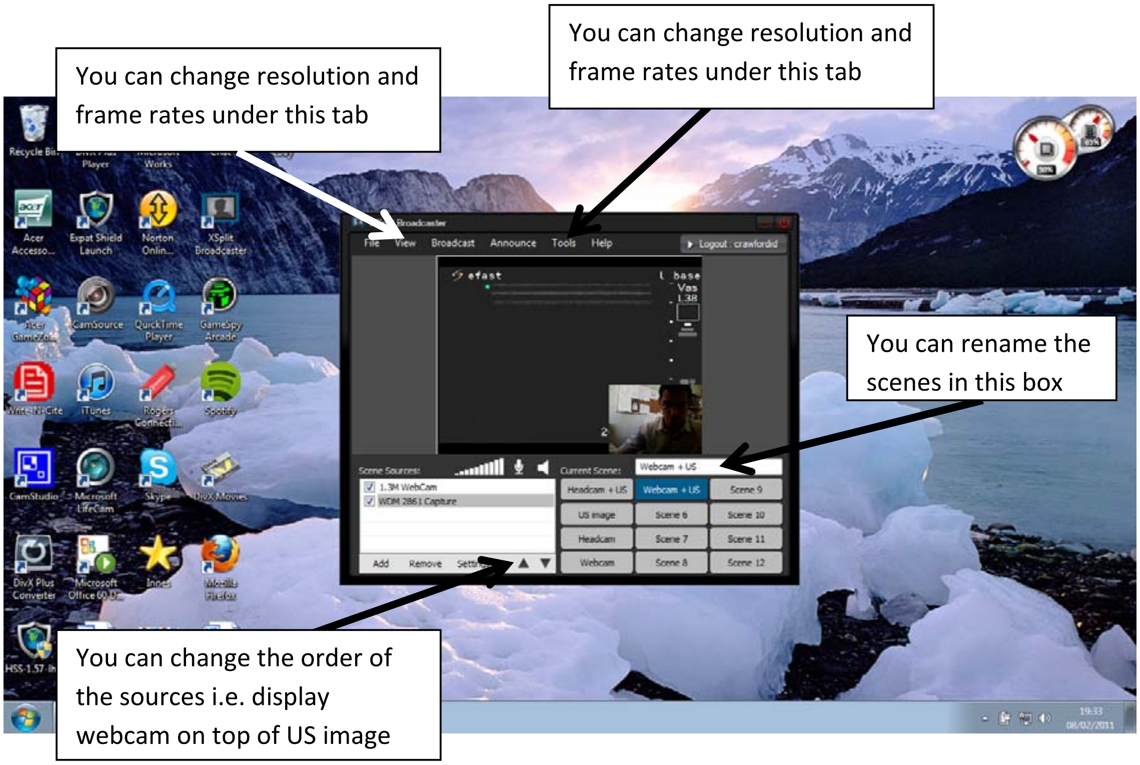

g. Calling

i. Double click on contact name in Skype (should have a green arrow next to their name to indicate they are online), this will then open up another window

ii. Click on videocall, this will send a call to the contact, the Video should automatically open up

2. **Subsequent use**
    a. Ensure webcam and videocapture (VC) devices are plugged into USB port of laptop and ensure VC device connected to video output on the US machine
    b. Connect to the internet before opening up XSplit
    c. Open up XSplit and ensure the appropriate scene and cameras are displaying, if not see 1.e.
    d. Open up Skype (normally connects automatically), double click on contact and click videocall
    e. Once finished ensure XSplit is closed before unplugging any devices

3. **Troubleshooting**
    a. No Video output is displayed when calling
        i. Check equipment on and connected properly
        ii. Check XSplit open and has appropriate scene and output selected
        iii. Check that Skype has XSplit selected as output under *'Video Settings'*(See 1.c.)
        iv. Check that when you are calling that the symbol □< is selected to transmit your video to the other user
    b. Call Quality is poor
        i. If you can select a Wireless Network at either or both ends, do so as the quality will be better
        ii. Get the remote expert to close down their video when calling, you don't really need to see their face, you just need to be able to hear them, this will free up some bandwidth
        iii. Check what laptop/smartphone you are using, most equipment from the health services will have security settings which can throttle the video quality, either take this up with IT or use your own laptop
    c. Everytime I load up XSplit I have to reselect the cameras in my scenes
        i. Ensure that before you open up XSplit everything is plugged in
        ii. Ensure you close XSplit before you unplug everything

    iii. Im hoping that XSplit will release an update to stop this but in the meantime the above should work

d. Only part of the US image is displayed when I make a videocall
    i. This is likely to be due to you having selected too high a resolution, try decreasing it, this should fix the problem
