# Supplementary material for: How to set up a low cost tele-ultrasound capable videoconferencing system with wide applicability
Source: Crit Ultrasound J. 2012 May 29;4(1):13. doi: 10.1186/2036-7902-4-13 (PMC3447660; doi:10.1186/2036-7902-4-13)
Supplement: Additional file 2 — Title: Making a video call. Description: Detailed instructions on how to initiate such tele-ultrasound sessions over a VOIP such as Skype (http://www.traumacanada.org/Default.aspx?pageId=829763). [file 2036-7902-4-13-S2.pdf]

Making a VideoCall

1. Ensure webcam and videocapture (VC) devices are plugged into USB port of laptop and ensure VC device connected to video output on the US machine
2. Connect to the internet before opening up XSplit
3. Open up XSplit and ensure the appropriate scene and cameras are displaying, if not see go to *Add* and *Add Camera* and select video sources you want to display (see 1.e. in Setup of VC system)

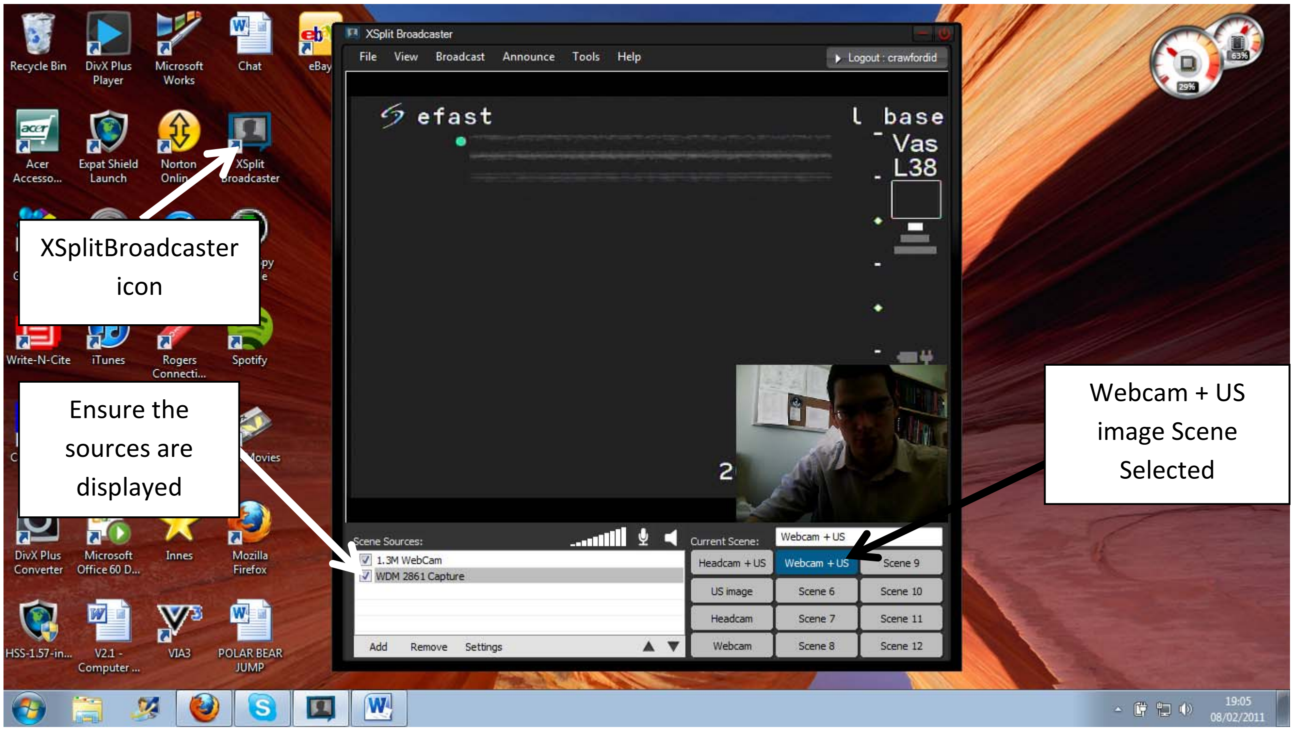

4. Open up Skype (normally connects automatically), double click on contact and click videocall

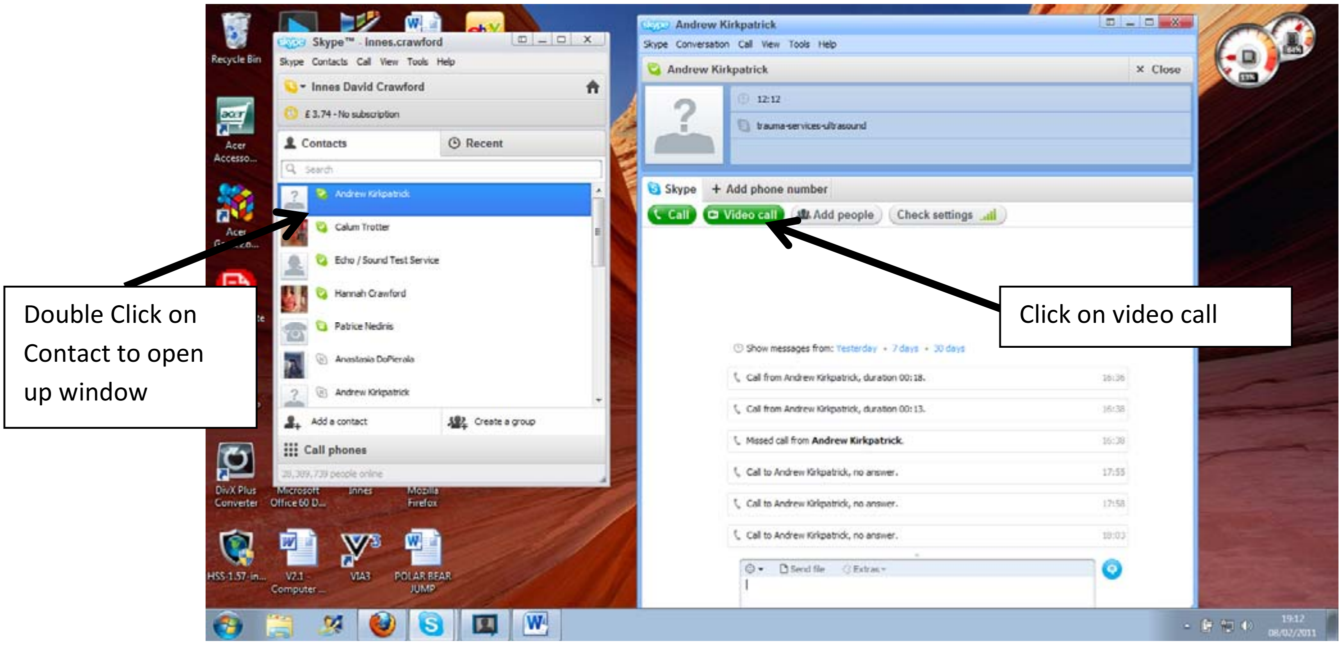

5. Once finished ensure XSplit is closed before unplugging any devices

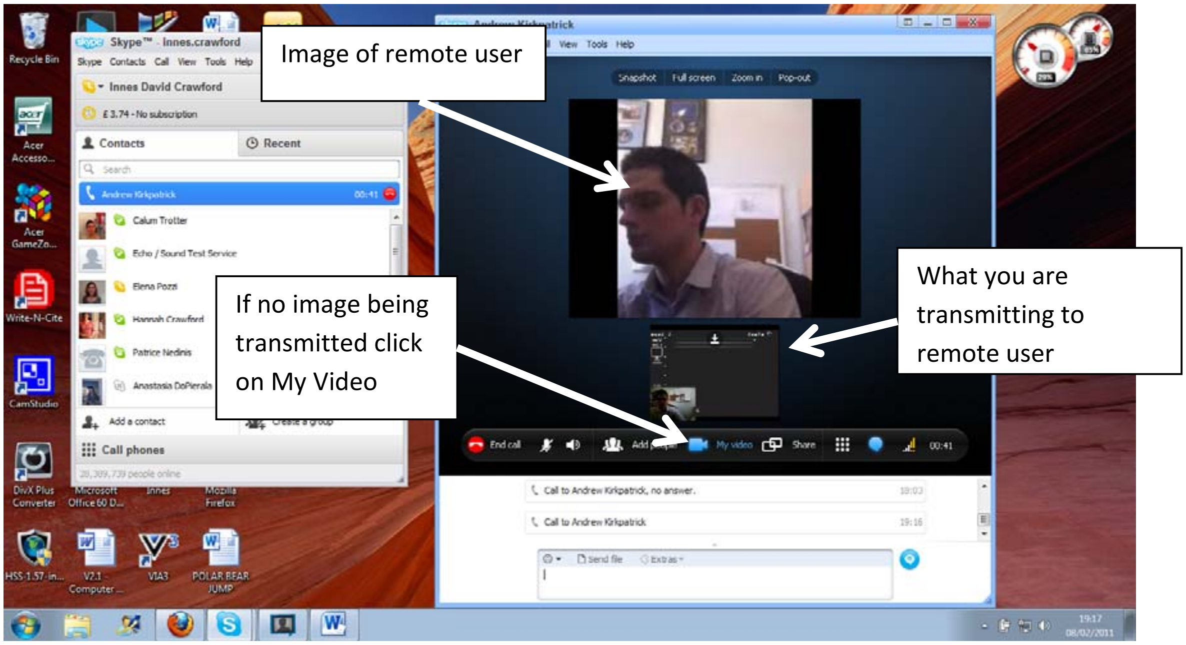

6. Once finished ensure XSplit is closed first before unplugging any devices
7. If any problems see troubleshooting
