# Supplementary material for: How to set up a low cost tele-ultrasound capable videoconferencing system with wide applicability
Source: Crit Ultrasound J. 2012 May 29;4(1):13. doi: 10.1186/2036-7902-4-13 (PMC3447660; doi:10.1186/2036-7902-4-13)
Supplement: Additional file 3 — Title: Additional file 3. Description: Tele-ultrasound recipe for the gourmets and aficionados (http://www.traumacanada.org). [file 2036-7902-4-13-S3.docx]

Tele-Ultrasound Recipe for the Gourmets and Afficianados

**Introduction:**

Ultrasound is a portable (often hand or pocket-carried) non-invasive, cost-effective, multidisciplinary/multisystem diagnostic and resuscitative tool that has potential applicability in nearly every aspect of patient care^1-3^. Tele-Ultrasound is a specific form of telemedicine that uses informatics advances to separate the patient from the expert interpreting the ultrasound findings, using techniques that are supported through tele-conferencing technology^4^. The teleconferencing equipment to facilitate this is currently “big business” as many health services in developed countries invest in it for the future. Videoconferencing (VC) equipment however can be notoriously expensive and can involve large start-up costs that discourage many health-care systems, as does the personal time commitment needed to respond to a call considering individual healthcare providers. Costs have been identified as one of the main barriers to the implementation of telemedicine particularly for developing countries who would likely benefit the most from this technology^5^.

Other informatics advances however, are furthering the revolution in connectivity and drastically reducing costs and logistic requirements. Voice over internet protocol (VOIP) allows voice and other multimedia (e.g. Video) to be transmitted via the internet instead of over other means like the public switched telephone network (PSTN) [normal telephone] or over Integrated service and digital network (ISDN) lines that are traditionally used for videoconferencing. VOIP is bandwidth efficient and has low running costs prompting many business’ including hospitals to migrate from using the traditional telephone to VOIP^6^. VOIP can also utilise cellular networks as well as wireless or Ethernet links allowing access wherever there is coverage. VOIP is also capable of linking into PTSN and mobile network providers allowing calls to these services^7^. Examples of VOIP providers include Apple’s - Facetime, Google’s Chat, Oovoo of New York, and one of the most widely used; Skype. Skype is a free VOIP service that currently provides low cost VC services to millions of people and businesses worldwide. With the continuing development of VOIP services, providers like Skype now offer many new services including group video-calling, HD videoconferencing and 3G video-calling^8^. VOIP, in particular Skype, has been investigated by several authors for use in healthcare mainly as a way of replacing older PSTN systems in hospitals to reduce costs^6^. It has also been investigated as a way of providing teaching in geographically difficult locations^9^ and for providing support in remote locations such as an Amazon swim expedition^10^ and for assessing Burn victims in Sao Tome^11^. While innovative, these prior applications only evaluated VOIP capabilities to provide basic videocalling and not for the transmission of other more robust forms of medical informatics. Recognizing a clinical need in both our own referral area, as well as globally, we thus demonstrated the ability to construct a low cost tele-ultrasound capable VC setup solution for the transmission of medical informatics over Skype^12,13^.

**Ingredients**

- **Equipment**: Two Commercially available laptop’s (£500-1000), Analogue to digital converter (£20), rocketstick/dongle (free with contract) and an Ultrasound machine (£ 2500 +)
- **Software**: VOIP software (Free), multimedia streaming software tool (Free)
- **People**: One Radiologist/sonographer (variable charge), One Remote operator (unsuspecting participant)

**Preparation Method**

The freely available VOIP software Skype (5.1) was installed onto modestly priced commercially available laptop computers (Acer Travelmate 5520 and Aspire 5741, Acer, Kuala Lumpur). A second, freely available software called XSplit Broadcaster (SplitMediaLabs ltd, Hong Kong) was concurrently installed, that allowed the display of several video inputs either simultaneously or independently by creating a virtual webcam, that could thereafter be selected as a video output on Skype. An inexpensive analogue to digital converter (VC-211V, ActionStar LinXcel, Taiwan) connected a modestly priced hand-carried ultrasound machine (NanoMaxx, Sonosite, Bothell, WA) to the laptop. A head- mounted webcam (LifeCam VX-2000, Microsoft, Washington) was also connected to the base-station laptop to provide real-time images of the patient and position of the ultrasound probe. Detailed “step-by-step” instructions on how to construct such a network may be accessed at (<http://www.traumacanada.org/Default.aspx?pageId=829763>)


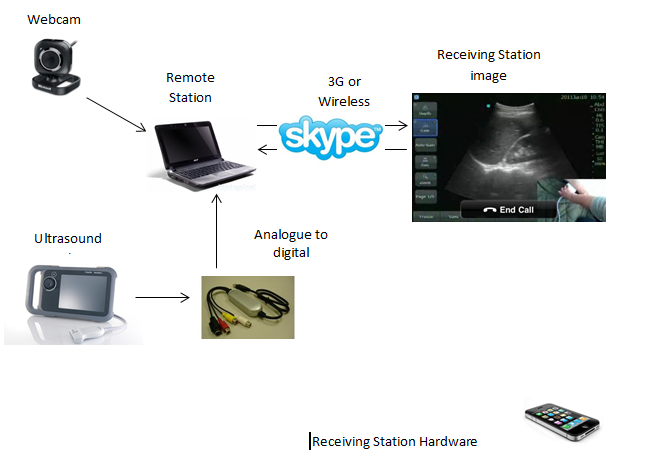


**To Check its taste**

A number of demonstrations verified the ability of this approach to facilitate remote mentored telesonography between geographically separate caregivers located on different continents, such as across the Atlantic between Calgary and alternatively Aberdeen, Scotland, and Rome and Pisa, Italy and in different remote environments, including from within small airplanes and from mountain-side locations^12,14^. Detailed instructions on how to initiate such tele-ultrasound sessions over Skype as an example of a VOIP may be accessed at (<http://www.traumacanada.org/Default.aspx?pageId=829763>).

Once such a system has been constructed to transmit from the site of the patient as a “package” delivered over VOIP, the receiving station may be as varied as there are computer or smart-phone devices that will support a particular VOIP software. This freedom allows the remote mentor to be able to use any of their desk-top computer, laptops, or Smart devices such as smart-phones or tablet type devices. For example, a video call was initiated by the user in Calgary, Canada to a panel of remote experts in Aberdeen, Scotland. The senders were connected to a 3G network, facilitated by use of a “dongle/rocketstick” (Rocket Mobile Internet Stick, Nokia Corp, Keilaniemi, Finland) to facilitate the connections between the laptops. A Rocketstick is essentially a device that allows the laptop to connect to the internet through the same way your smartphone does when not connected to Wifi. Your smartphone and Tablet computers normally have this built in, and you can in fact use some mobiles themselves as a “rocketstick”. The remote experts then guided a user through both a basic Focused Assessment with Sonography for Trauma (FAST^15^) performed on an ultrasound phantom and a volunteer, interpreting the images as they were captured^13^. The Extended FAST exam^15^ examining the pleural spaces was also performed and demonstrated remotely^16^.
